# Biodiversity insights from BioBlitz Surveys on Terceira Island, Azores

**DOI:** 10.3897/BDJ.13.e153461

**Published:** 2025-05-07

**Authors:** Paulo A. V. Borges, Jagoba Malumbres-Olarte, Rosalina Gabriel, Sandra I. R. Videira, António Félix Rodrigues, Sébastien Lhoumeau, Abrão Leite, Alejandra Ros-Prieto, Cecília Melo, Gabor Pozsgai, Guilherme Oyarzabal, Laurine Parmentier, Lucas Lamelas-López, Mário Boieiro, Paulo J. M. Barcelos, Ricardo Costa, Rúben Coelho, Sophie Wallon, Susana Gonçalves, Ana M. Arroz, Isabel R. Amorim

**Affiliations:** 1 University of the Azores, cE3c- Centre for Ecology, Evolution and Environmental Changes/Azorean Biodiversity Group, CHANGE – Global Change and Sustainability Institute, School of Agricultural and Environmental Sciences, Rua Capitão João d´Ávila, Pico da Urze, 9700-042, Angra do Heroísmo, Azores, Portugal University of the Azores, cE3c- Centre for Ecology, Evolution and Environmental Changes/Azorean Biodiversity Group, CHANGE – Global Change and Sustainability Institute, School of Agricultural and Environmental Sciences, Rua Capitão João d´Ávila, Pico da Urze, 9700-042 Angra do Heroísmo, Azores Portugal; 2 IUCN SSC Atlantic Islands Invertebrate Specialist Group, Angra do Heroísmo, Azores, Portugal IUCN SSC Atlantic Islands Invertebrate Specialist Group Angra do Heroísmo, Azores Portugal; 3 IUCN SSC Monitoring Specialist Group, Angra do Heroísmo, Azores, Portugal IUCN SSC Monitoring Specialist Group Angra do Heroísmo, Azores Portugal; 4 LIBRe – Laboratory for Integrative Biodiversity Research, Finnish Museum of Natural History, University of Helsinki, P.O.Box 17 (Pohjoinen Rautatiekatu 13), 00014, Lisboa, Portugal LIBRe – Laboratory for Integrative Biodiversity Research, Finnish Museum of Natural History, University of Helsinki, P.O.Box 17 (Pohjoinen Rautatiekatu 13), 00014 Lisboa Portugal; 5 Fundação Gaspar Fructuoso, Angra do Heroísmo, Azores, Portugal Fundação Gaspar Fructuoso Angra do Heroísmo, Azores Portugal; 6 IITAA – University of the Azores, Instituto de Investigação e Tecnologias Agrárias e do Ambiente, Angra do Heroísmo, Azores, Portugal IITAA – University of the Azores, Instituto de Investigação e Tecnologias Agrárias e do Ambiente Angra do Heroísmo, Azores Portugal; 7 Rua Fernando Pessoa, nº99 R/C DTO 2765-483, Estoril, Portugal Rua Fernando Pessoa, nº99 R/C DTO 2765-483 Estoril Portugal; 8 Quinta da Vinagreira, São Bartolomeu 9700-518, Angra do Heroísmo, Azores, Portugal Quinta da Vinagreira, São Bartolomeu 9700-518 Angra do Heroísmo, Azores Portugal; 9 University of the Azores, cE3c- Centre for Ecology, Evolution and Environmental Changes/Azorean Biodiversity Group, CHANGE – Global Change and Sustainability Institute, Rua Capitão João d´Ávila, Pico da Urze, 9700-042, Angra do Heroísmo, Azores, Portugal University of the Azores, cE3c- Centre for Ecology, Evolution and Environmental Changes/Azorean Biodiversity Group, CHANGE – Global Change and Sustainability Institute, Rua Capitão João d´Ávila, Pico da Urze, 9700-042 Angra do Heroísmo, Azores Portugal; 10 Associação Os Montanheiros, Rua da Rocha, 8, 9700-169, Angra do Heroismo, Azores, Portugal Associação Os Montanheiros, Rua da Rocha, 8, 9700-169 Angra do Heroismo, Azores Portugal; 11 Secretaria Regional do Ambiente e Alterações Climáticas, Rua do Galo nº 118, 9700-040, Angra do Heroísmo, Azores, Portugal Secretaria Regional do Ambiente e Alterações Climáticas, Rua do Galo nº 118, 9700-040 Angra do Heroísmo, Azores Portugal; 12 Câmara Municipal de Angra do Heroísmo, Praça Velha, Angra do Heroísmo, Azores, Portugal Câmara Municipal de Angra do Heroísmo, Praça Velha Angra do Heroísmo, Azores Portugal

**Keywords:** biodiversity, citizen science, historic garden, lichens, vascular plants, arthropods, birds

## Abstract

**Background:**

This manuscript is the first scientific publication of the project “BioBlitz Azores". The project was launched in 2019 and had a second event in 2023 under the scope of the FCT-MACRISK project, surveying the historic public garden "Jardim Duque da Terceira", in the historical centre of Angra do Heroísmo, Terceira Island (Azores, Portugal). In addition to contributing directly to the knowledge of Azorean biota, BioBlitz Azores aims to engage the non-scientific community - including volunteers, amateur naturalists, students, teachers, families and other garden visitors - to foster a sense of community and raise awareness about Azorean biodiversity and its conservation.

**New information:**

Under the scope of two BioBlitz events, the list of taxa of the historic garden of "Jardim Duque da Terceira" (Terceira, Azores, Portugal) was updated and presently includes 72 lichen species, 55 vascular plant species, 96 arthropod species, 14 bird species and three freshwater vertebrate species.

In the realm of lichens, two species are new records for Portugal and Macaronesia, one species is a new record for the Azores and nine species are new records for Terceira Island. This is the first academic publication for 11 of the 12 lichen species.

The survey of arthropods yielded an inventory encompassing a total of 96 taxa, with 78 of these identified to the species or subspecies level; amongst the identified taxa, three are endemic, 32 are native, but not endemic, one is of indeterminate origin and 42 are introduced. Notably, a single specimen of the rare endemic spider, *Savigniorrhipisacoreensis* Wunderlich, 1992 was observed for the first time at this low elevation (garden elevation: 29-60 m a.s.l.). The species is typically found in the canopies of endemic trees species in native forests at mid- to high elevations (500-1000 m a.s.l.) and its presence in the garden suggests a source-sink dynamic of this extremely dispersive species between native and anthropogenic habitats.

Regarding vascular plants, 54 taxa were recorded in the garden, comprising one endemic, one native, three with indeterminate origin and 49 introduced ornamental species.

Amongst birds, 14 taxa were registered, including seven Azorean endemic subspecies, two native species and four introduced taxa.

Three freshwater vertebrate species were recorded during the survey, all of which are exotic species that have been introduced to the garden.

## Introduction

Bioblitz events provide a valuable platform for biodiversity assessment, general public engagement and conservation action, making them a powerful tool to understand and protect the natural world (Parker et al. 2018, Meeus et al. 2023). The Bioblitz concept was first developed in 1996 by the National Park Service in the United States, in which scientists and the public conducted an intensive survey of the biodiversity at the Kenilworth Aquatic Gardens within a 24-hour period, thus setting the model for future BioBlitz events (Ruch et al. 2010). This kind of events are now common and provide several benefits, the most important being arguably public involvement in biodiversity surveys (Parker et al. 2018, Meeus et al. 2023, Palma et al. 2024). Bioblitz events promote non-scientific public-engagement with biodiversity, but are also currently used as a baseline for biodiversity monitoring, helping to track changes over time and provide information for conservation strategies (Palma et al. 2024). More importantly, these events can build stronger connections within the local community, encouraging ongoing collaboration and support for biodiversity initiatives and be a powerful tool for environmental education (Páez-Vacas et al. 2023). Following several of the "Partners" principle (Mishra et al. 2017), which encourage building strong relationships with local people (presence effect), bioblitzes invest in attracting new public (aptness principle), engage in open and honest communication (respect principle) and act as a bridge between local communities and wildlife experts and managers (strategic support principle).

Given the recognition of an increasing disconnection between people and nature (Soga and Gaston 2023), bioblitzes may foster pro-environmental attitudes and behaviour (Dean et al. 2018) and establish long-term engagement and advocacy for biodiversity stewardship (Gass et al. 2021). Bioblitzes may also often attract media coverage, which can further raise awareness about biodiversity issues and promote conservation messages to a broader audience (Francis et al. 2017).

Importantly, BioBlitz events, when combined with digital platforms and apps, can contribute to larger databases, such as GBIF (Global Biodiversity Information Facility) and iNaturalist (Biodiversity4All - Portuguese platform), enhancing global biodiversity records and research (Aristeidou et al. 2021).

In Portugal, bioblitzes are organised periodically since 2013, following the first event led by the Serralves Foundation in Oporto. This event typically involves collaboration with universities, research institutions and environmental organisations, featuring a variety of activities, including species identification workshops, guided tours and educational sessions for all ages (de Vasconcelos Monteiro 2015). Other BioBlitz events occur in Oeiras and Lisbon, often involving local schools, universities and environmental groups, but also aiming to catalogue urban and peri-urban biodiversity and raising awareness about the natural richness within the city (Tiago et al. 2024). Some events focus on particular groups, such as plants (Chozas et al. 2023, Tiago et al. 2024), pollinators (Fontúrbel et al. 2024) or freshwater microinvertebrates (Laforest et al. 2013), others encompass a broader taxonomic range (see revision in Meeus et al. (2023)), but all provide valuable biodiversity data.

Being part of the Mediterranean biodiversity hotspot (Myers et al. 2000, Neff 2001) and the Macaronesia biogeographical region (Fernández‐Palacios et al. 2024), the Azorean Archipelago, located in the North Atlantic Ocean, is of significant biodiversity importance due to its unique combination of geographic isolation, varied habitats and high levels of endemism (Borges et al. 2020, Borges et al. 2022). The Archipelago features a wide range of habitats, including several types of native forests (Elias et al. 2016) that are now restricted to mid- and high elevations and are threatened by the impact of exotic species (Borges et al. 2006, Borges et al. 2020) and climatic changes (Ferreira et al. 2016).

Low elevation habitats are mostly anthropogenic and highly disturbed, not only by urbanisation, but also by the introduction of exotic species (Barreiros et al. 2010, Borges et al. 2013, Lamelas-López et al. 2023, Boieiro et al. 2024). However, recent evidence shows that some Azorean lowland-endemic arthropod species are still present at low isolated forest patches (Tsafack et al. 2021).

Parks and gardens, often located in urban areas at low elevation, may serve as sentinels both for the introduction of new alien species - often coming from ports and airports near the coast, while contributing to the safeguarding of indigenous non-target species due to the high humidity and great diversity of substrates available. Thus, complementary species, such as insects, spiders, lichens and bryophytes, may thrive in gardens, contributing to increasing the ecological complexity of those areas. These spaces may provide shelter from anthropogenic pressures, benefitting rare and/or threatened species. In fact, some historic gardens in the Azores have proven to be quite rich in arthropod species (e.g. Arteaga et al. (2020), Lamelas-López et al. (2023)), while different recreational parks succeed in increasing the bryophyte diversity of the Region (e.g. Polaino-Martín et al. (2020)). Concomitantly, it is also true that many invasive alien species, presently occurring in the Azores and elsewhere, were originally ornamental plant species that escaped gardens and parks (Gabriel 2019).

Therefore, BioBlitz events conducted in low-elevation habitats, such as the public garden in Angra do Heroísmo (29-60 m a.s.l.), are expected to provide novel data on the presence and distribution of rare endemic species on one hand, while also improving the data on recently introduced exotic species' distributions.

## General description

### Purpose

The main objective of this publication is to share the results of the BioBlitz multi-taxa inventories in the “Jardim Duque da Terceira” in Angra do Heroísmo (Terceira Island, Azores, Portugal) that took place in 2019 and in 2023. Beyond documenting the rich biodiversity of this unique location, this publication aims also to:

- Inspire local and global communities to engage in citizen-science and biodiversity monitoring initiatives.
- Encourage policy-makers, researchers and conservationists to prioritise the improvement of urban habitats for biodiversity conservation.
- Serve as an educational resource, demonstrating the value of collaborative efforts amongst scientists, citizens and educators in exploring and protecting natural heritage.
- Highlight the cultural and scientific importance of integrating historic gardens like “Jardim Duque da Terceira” into conservation strategies.

Thus, this publication aspires to contribute to the broader goals of biodiversity research, environmental education and the sustainable management of urban green spaces, contributing to biodiversity conservation.

## Project description

### Title

BioBlitz Azores: Multitaxa inventories of the biodiversity of “Jardim Duque da Terceira” (Duke of Terceira Garden, Angra do Heroísmo,Terceira Island, Azores, Portugal)

### Personnel

The project was conceived and is being led by Isabel R. Amorim and Jagoba Malumbres-Olarte.

Fieldwork (site selection and experimental setting): António Félix Rodrigues, Cecília Melo, Isabel R. Amorim, Jagoba Malumbres-Olarte, Lucas Lamelas-López, Paulo Barcelos, Paulo A. V. Borges, Rúben Coelho, Susana Gonçalves.

Fieldwork (authorisation): José Álamo Meneses (Mayor of Angra do Heroísmo).

Fieldwork (Higher taxa coordination): The lichen inventory was coordinated by António Félix Rodrigues; the vascular plants inventory was coordinated by Susana Gonçalves and Paulo J.M. Barcelos; the arthropod inventory was coordinated by Paulo A.V. Borges; the bird inventory was coordinated by Cecília Melo and Rúben Coelho. In the 2019 BioBlitz Azores, the freshwater invertebrate survey was led by Lucas Lamelas-López.

Fieldwork (Trainers in place): Abrão Leite, Alejandra Ros-Prieto, António Félix Rodrigues, Gabor Pozsgai, Guilherme Oyarzabal, Isabel R. Amorim, Jagoba Malumbres-Olarte, Mário Boieiro, Paulo A.V. Borges, Paulo J.M. Barcelos, Paulo Mendonça, Ricardo Costa, Rúben Coelho, Sébastien Lhoumeau, Sophie Wallon, Susana Gonçalves, Cecília Melo.

Parataxonomists (Laboratory): ARTHROPODA - Abrão Leite, Alejandra Ros-Prieto, Laurine Parmentier.

Taxonomists: António Félix Rodrigues and Rosalina Gabriel (lichens); Paulo A.V. Borges (arthropods); Lucas Lamelas-López (freshwater organisms); Susana Gonçalves and Paulo J.M. Barcelos (vascular plants); Cecília Melo and Rúben Coelho (birds).

Arthropod Curation: Voucher specimen management was mainly undertaken by Alejandra Ros-Prieto, Abrão Leite, Ricardo Costa and Paulo A. V. Borges.

Lichens Curation: Voucher specimen management was undertaken by António Félix Rodrigues.

Darwin Core Databases: Paulo A.V. Borges, Sébastien Lhoumeau, Sandra Videira, Rosalina Gabriel.

### Study area description

This study was conducted in Angra do Heroísmo, on Terceira Island (Azores, Portugal).

Terceira Island (total area: 400.2 km²; maximum elevation: 1021 m above sea level) is part of the central group of the Azores Archipelago in the North Atlantic, located approximately at coordinates 38°43′40″N, 27°12′48″W. The climate of the Azores Archipelago is temperate oceanic, characterised by regular and abundant rainfall, high levels of relative humidity and persistent western winds (Forjaz 2004). The landscape of the islands is predominantly urban and agricultural at lower elevations, with pasturelands and exotic tree plantations inland and native forests at higher elevations (Elias et al. 2016).

**Figure 1.**
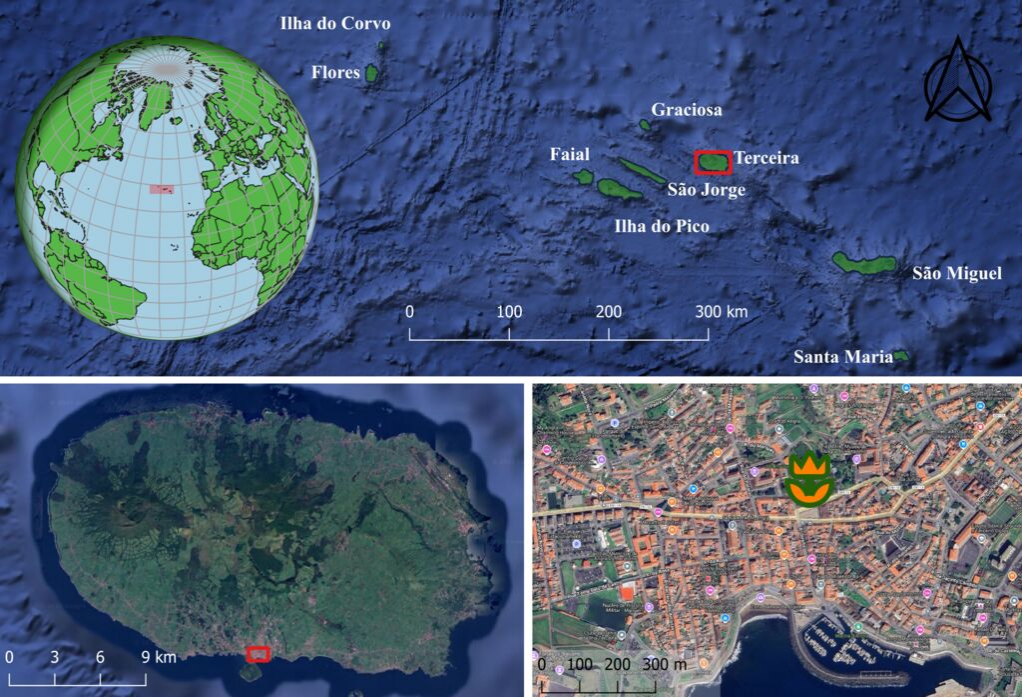
The location of the Garden "Jardim Duque da Terceira" in Terceira Island (Azores) (Credit: Gabor Pozsgai).

The "Jardim Duque da Terceira" (Fig. 1) is a public historic garden located in the centre of Angra do Heroísmo, the largest city of Terceira Island. This garden is named after the Duke of Terceira, a hero of the Liberal Wars (1832-1834), a title commemorating the island's historical significance and contribution to history. Established on 18 January 1888, the "Jardim Duque da Terceira" features a mix of exotic plant species from around the world (Arteaga et al. 2020, Lamelas-López et al. 2023), most of them with informative plates regarding their taxonomy and biogeographic origin. The layout of "Jardim Duque da Terceira" features winding paths and distinct thematic sections, including rose gardens, tropical plant collections and shaded groves. This garden is both a botanical treasure and a cultural and historical landmark of Angra do Heroísmo City.

### Design description

During the BioBlitz Açores, both in 2019 (Malumbres-Olarte et al. 2019) and 2023 (Amorim et al. 2023, Borges et al. 2023), a range of targeted and specialised sampling protocols were employed to assess biodiversity across different taxa (see below).

Each session was about two hours long and participants could choose their area/taxa of interest beforehand: lichens, arthropods, freshwater organisms, birds and vascular plants. The sessions began with a briefing to explain the process and goals of the BioBlitz, setting expectations on what participants would learn and how they would contribute to local biodiversity knowledge. In addition to field observations, a mini-laboratory was set up in the garden where participants could use binocular sterereomicroscopes and hand lenses to examine finer details of specimens, which are crucial for the identification of smaller species like insects, spiders or lichens. The combination of in situ observations with subsequent laboratory work is a well-established and complementary method in biodiversity assessments. Laboratory work allows for detailed taxonomic verification. Preserved specimens serve as vouchers that can be re-examined, compared against reference collections and used for DNA barcoding, ensuring robustness in species identification. The sampling was conducted under the necessary permits and ethical guidelines. The number of specimens collected was minimised to balance scientific needs with conservation imperatives.

### Funding

Azorean Regional Secretariat for the Sea, Science and Technology; Azorean Regional Directorate of Science and Technology - BioBLitz Azores (DRCT M3.4.B/CIÊNCIA CIDADÃ/004/2019/RTF/033).

Science and Technology Foundation (FCT) - MACRISK-Trait-based prediction of extinction risk and invasiveness for Northern Macaronesian arthropods (FCT-PTDC/BIA-CBI/0625/2021).

Portal da Biodiversidade dos Açores (2022-2023) - PO Azores Project - M1.1.A/INFRAEST CIENT/001/2022 (2022).

FCT-UIDB/00329/2020-2024, DOI 10.54499/UIDB/00329/2020 (Thematic Line 1–integrated ecological assessment of environmental change on biodiversity).

FCT-UID/00329/2025 - Centre for Ecology, Evolution and Environmental Changes (CE3C).

Azores DRCT Pluriannual Funding (M1.1.A/FUNC.UI&D/010/2021-2024).

IRA and MB were funded by Portuguese national funds through FCT – Fundação para a Ciência e a Tecnologia, I.P., under the Norma Transitória https://doi.org/10.54499/DL57/2016/CP1375/CT0003 and https://doi.org/10.54499/DL57/2016/CP1375/CT0004.

## Sampling methods

### Sampling description

Lichens: The search for lichens mainly involved visual inspection of tree bark and rocks. Participants occasionally collected samples for closer examination under magnification tools to accurately identify the species.

Vascular Plants: Participants examined various plant features such as size, leaves, flowers and fruit details and, in some cases, utilised their senses of smell and touch to aid in species identification.

Arthropods: Different capture and observation techniques were applied depending on the habitat and the type of arthropods being studied. These techniques were explained in detail to participants before the start of the session. Two main methods were used: a) **Sweep Netting** through which participants used sweep nets to collect arthropods from vegetation (involving sweeping a net through the foliage where arthropods might be resting or feeding, which is effective for catching flying or jumping insects); and b) **Beat Sampling**, which was used to dislodge arthropods from trees and bushes. For the latter, participants held a sheet or tray under a branch and then shook or beat the branch, causing arthropods to fall on to the sheet for collection and identification.

Birds: Birdwatching required participants to be quiet and observant, using binoculars and listening for bird calls to locate and identify species both in the tree canopy and on the ground.

Freshwater organisms: Fish and amphibians observations required the participants to quietly observe the water stream and pools in several locations of the garden.

### Quality control

Species taxonomic nomenclature for arthropods follows Borges et al. (2022). For lichens several sources were followed (Aptroot et al. 2010, Lücking et al. 2017a, Lücking et al. 2017b). Concerning vascular plants, we followed Silva et al. (2010) and, for birds and amphibians, we followed Rodrigues et al. (2010).

## Geographic coverage

### Description

This study was conducted in a city public garden "Jardim Duque da Terceira" in Angra do Heroísmo on Terceira Island (Azores, Portugal).

### Coordinates

38.655 and 38.661 Latitude; -27.223 and -27.213 Longitude.

## Taxonomic coverage

### Taxa included

**Table:** 

Rank	Scientific Name	Common Name
phylum	Ascomycota	Lichens
phylum	Ginkgophyta	Ginkgo
phylum	Pteridophyta	Ferns
phylum	Pinophyta	Conifers
phylum	Magnoliophyta	Flowering plants
phylum	Arthropoda	Arthropods
class	Actinopterygii	Fish
class	Amphibia	Frog
class	Aves	Birds

## Temporal coverage

### Notes

BioBlitz Azores was conducted on 27 July 2019 and 17 June 2023.

## Collection data

### Collection name

For the collected arthropods - Entomoteca Dalberto Teixeira Pombo at University of the Azores.

### Collection identifier

DTP

### Specimen preservation method

Alcohol.

## Usage licence

### Usage licence

Creative Commons Public Domain Waiver (CC-Zero)

## Data resources

### Data package title

BioBlitz Azores: Multitaxa inventories of the biodiversity of “Jardim Duque da Terceira” (Angra do Heroísmo, Terceira Island, Azores, Portugal).

### Resource link

https://doi.org/10.15468/eqc6n5

### Alternative identifiers

https://www.gbif.org/dataset/1c3fa6fb-4242-461a-ba66-742143b3ae57

### Number of data sets

2

### Data set 1.

#### Data set name

Event Table

#### Data format

Darwin Core Archive

#### Character set

UTF-8

#### Download URL

http://ipt.gbif.pt/ipt/resource?r=bioblitz_terceira

#### Data format version

1.5

#### Description

The dataset was published in the Global Biodiversity Information Facility platform, GBIF (Borges et al. 2025). The following data table includes all the records for which a taxonomic identification of the species was possible. The dataset submitted to GBIF is structured as a sample event dataset that has been published as a Darwin Core Archive (DwCA), which is a standardised format for sharing biodiversity data as a set of one or more data tables. The core data file contains 58 records (eventID). This GBIF IPT (Integrated Publishing Toolkit, Version 2.5.6) archives the data and, thus, serves as the data repository. The data and resource metadata are available for download in the Portuguese GBIF Portal IPT (Borges et al. 2025).

**Data set 1.** 

Column label	Column description
id	Unique identification code for sampling event data.
type	The nature or genre of the resource, as defined by the Dublin Core standard. In our case "PhysicalObject" or "Event".
datasetName	The name (or acronym) in use by the institution having ownership of the object(s) or information referred to in the record. In our case, we use different names for each taxonomic group.
eventID	Identifier of the events, unique for the dataset.
samplingProtocol	The sampling protocol used to capture or observe the species.
sampleSizeValue	The numeric amount of time spent in each sampling.
sampleSizeUnit	The unit of the sample size value.
eventDate	Range during which the record was collected.
year	The four-digit year in which the dwc:Event occurred, according to the Common Era Calendar.
month	The integer month in which the dwc:Event occurred.
day	The integer day of the month on which the dwc:Event occurred.
habitat	The habitat from which the sample was obtained.
locationID	Identifier of the location.
continent	The name of the continent in which the dcterms:Location occurs (Europe).
islandGroup	Name of archipelago, always Azores in the dataset.
island	Name of the island, always Terceira in the dataset.
country	Country of the sampling site, always Portugal in the dataset.
countryCode	ISO code of the country of the sampling site, always PT in the dataset.
municipality	Municipality of the sampling site, always Angra do Heroísmo in the dataset.
locality	Name of the locality, always Angra do Heroísmo in the dataset.
minimumElevationInMetres	The lower limit of the range of elevation (altitude, above sea level), in metres.
decimalLatitude	Approximate decimal latitude.
decimalLongitude	Approximate decimal longitude.
geodeticDatum	The ellipsoid, geodetic datum or spatial reference system (SRS), upon which the geographic coordinates given in decimalLatitude and decimalLongitude are based, always WGS84 in the dataset.
coordinateUncertaintyInMetres	Uncertainty of the coordinates of the centre of the sampling plot.
coordinatePrecision	Precision of the coordinates.
georeferenceSources	A list (concatenated and separated) of maps, gazetteers or other resources used to georeference the Location, described specifically enough to allow anyone in the future to use the same resources.
fieldNumber	Code for the sample.

### Data set 2.

#### Data set name

Occurrence Table

#### Data format

Darwin Core Archive

#### Character set

UTF-8

#### Download URL

http://ipt.gbif.pt/ipt/resource?r=bioblitz_terceira

#### Data format version

1.5

#### Description

The dataset was published in the Global Biodiversity Information Facility platform, GBIF (Borges et al. 2025). The following data table includes all the records for which a taxonomic identification of the species was possible. The dataset submitted to GBIF is structured as an occurrence table that has been published as a Darwin Core Archive (DwCA), which is a standardised format for sharing biodiversity data as a set of one or more data tables. The core data file contains 490 records (occurrenceID). This GBIF IPT (Integrated Publishing Toolkit, Version 2.5.6) archives the data and, thus, serves as the data repository. The data and resource metadata are available for download in the Portuguese GBIF Portal IPT (Borges et al. 2025).

**Data set 2.** 

Column label	Column description
id	Unique identification code for sampling event data.
licence	Reference to the licence under which the record is published.
institutionID	The identity of the institution publishing the data.
collectionID	The identity of the collection publishing the data.
institutionCode	The code of the institution publishing the data.
collectionCode	The code of the collection where the specimens are conserved.
basisOfRecord	The nature of the data record.
occurrenceID	Identifier of the record, coded as a global unique identifier.
recordedBy	A list (concatenated and separated) of names of people, groups or organisations who performed the sampling in the field.
organismQuantity	A number or enumeration value for the quantity of organisms.
organismQuantityType	The type of quantification system used for the quantity of organisms.
establishmentMeans	The process of establishment of the species in the location, using a controlled vocabulary: 'native', 'introduced', 'endemic', 'indeterminate'.
occurrenceRemarks	Comments or notes about the dwc:Occurrence, namely the substrate in which some lichens were found.
eventID	Identifier of the events, unique for the dataset.
identifiedBy	A list (concatenated and separated) of names of people, groups or organisations who assigned the taxon to the subject.
dateIdentified	The date on which the subject was determined as representing the taxon.
identificationRemarks	Comments or notes about the dwc:Identification. We mention the AZORES BIOPORTAL code for the vascular plants and vertebrates and the Morphspecies code for the arthropods.
scientificName	Complete scientific name including author and year.
kingdom	Kingdom name.
phylum	Phylum name.
class	Class name.
order	Order name.
family	Family name
genus	Genus name.
specificEpithet	Specific epithet.
infraspecificEpithet	Infraspecific epithet.
taxonRank	Lowest taxonomic rank of the record.
scientificNameAuthorship	Name of the author of the lowest taxon rank included in the record.

## Additional information

In the surveys across several taxonomic groups, a total of 240 taxa was documented, including 221 species or subspecies level identifications.

In the realm of lichens, 72 taxa were identified, highlighting both their ecological significance and diversity. In the past decades, the known diversity of lichens in the Azores has been steadily increasing, reflecting a growing comprehension of this important group in the Archipelago (Aptroot et al. 2009, Aptroot et al. 2010, Rodrigues et al. 2024, Rodrigues and Aptroot 2024). Several species are new records for Terceira (nine species), Azores (one species) and Portugal and Macaronesia (two species) (see Table 1).

*Verseghyathysanophora* (R.C.Harris) S.Y.Kondr., is a leprose, crustose lichen, with a thallus characterised by a thin, patchy layer of granular soredia, ranging in colour from pale green to yellowish-green, often encircled by a conspicuous white, fibrous prothallus. It was originally described under the genus *Lecanora.* The lichen is widely distributed across the Northern Hemisphere, usually growing on bark of deciduous trees; however, in "Jardim Duque da Terceira" (Angra do Heroísmo, Terceira, Azores), it was found colonising a rocky wall. *Biatoraefflorescens* (Hedl.) Räsänan is a crustose lichen, with a granular, greyish to green thallus. This lichen has a Northern Hemisphere distribution and is mainly found on forests, growing on non-saxicolous substrates; indeed, in Terceira Island, it was found colonising a tree. Both lichens are new records for Portugal and Macaronesia.

The first record of *Leprariamembranacea* (Dicks.) Vain. in the Azores was documented during the 2023 BioBlitz event (Amorim et al. 2023) and it has subsequently been cited from two additional locations in Terceira Island (Rodrigues and Aptroot 2024). It is a leprose, crustose lichen characterised by a pale yellowish to cream-coloured thallus, that forms well-defined, lobed, membrane-like rosettes. It is a cosmopolitan lichen species, with widespread distribution across Europe and North America. It generally grows on acidic rocks and, in Terceira Island, was found growing on lapilli.

Finally, according to the latest Azorean checklist (Aptroot et al. 2010) and the Azorean Biodiversity Portal (ABP 2024), nine species are new records for Terceira Island: *Diplotommaambiguum* (Ach.) Flagey, previously known from Faial Island; *Fuscopannarianebulosa* (Hoffm.) E.Tripp & Lendemer, previously known from Pico and Faial Islands; *Hypocenomycescalaris* (Ach. ex Lilj.) M.Choisy (Fig. 2b) and *Opegraphavermicellifera* (J.Kunze) J.R.Laundon, previously known from São Miguel Island; *Hypotrachynarevoluta* (Flörke) Hale (Fig. 2c) and *Physciacaesia* (Hoffm.) Fürnr., previously known from São Miguel Island and recently observed also in Corvo Island (Rodrigues et al. 2024); *Polycaulionacandelaria* (Linnaeus) Frödén, Arup & Søchting (Fig. 2f), previously known from Pico and São Jorge Islands; *Pertusariapertusa* (L.) Tuck. (Fig. 2d), previously known from Faial and São Miguel Islands; and *Pseudoschismatommarufescens* (Pers.) Ertz & Tehler (Fig. 2e), previously known from Graciosa Island.

In addition, the species *Botryoleprarialesdainii* (Hue) Canals, Hern.-Mariné, Gómez-Bolea & Llimona (Fig. 2a) was observed for the first time in Terceira Island during the BioBlitz event in 2023 (Amorim et al. 2023) and it has subsequently been cited from two additional locations in the Island (Rodrigues and Aptroot 2024). The crustose lichen *Mycoblastusaffinis* (Schaer.) T.Schauer, had been referred to Terceira Island without any precise location by Aptroot et al. (2010) and its presence is now confirmed for the "Jardim Duque da Terceira", Angra do Heroísmo, Terceira, Azores.

Regarding vascular plants, 54 taxa were distinguished, comprising 52 identified at species level - including one endemic, one native, one with indeterminate origin and 49 introduced species (Table 2). This mix emphasises the influence of both native and non-native species on the local flora, dominated in this garden by exotic species (Arteaga et al. 2020, Lamelas-López et al. 2023). Despite being dominated by exotic species, gardens play crucial ecological and human health/well-being functions. Even if the species are not native, they can offer necessary shelter and food for local fauna, contributing to urban biodiversity. Indeed, in this public garden, vascular plants enhance biodiversity by structuring habitats and providing resources for a variety of wildlife, including lichens, bryophytes, arthropods and birds. Moreover, the diversity of plant species in a public garden can serve as a living library that promotes education about different flora from around the world. Thus, notwithstanding the invasive potential, that needs to be assessed, exotic vascular plants can have practical uses in research and education, providing opportunities for botanical studies and supporting programmes that teach about plant taxonomy, biology, ecology and conservation.

The survey of arthropods yielded an inventory encompassing a total of 96 taxa, with 78 of these identified to the species or subspecies level (Table 3). Our findings included three endemic taxa, 32 native, one of indeterminate origin and 42 introduced taxa. Notably, we observed the presence of the rare endemic spider, *Savigniorrhipisacoreensis* Wunderlich, 1992 (Araneae, Linyphiidae) (Fig. 3a). This species is typically restricted to the canopies of endemic trees within native forests at mid- to high elevations. Based on the species’ life-history traits (Macías-Hernández et al. 2020) and its widespread distribution across several islands in the Azores (Borges et al. 2022, Pozsgai et al. 2024), this specimen may be indicative of a source–sink dynamic that facilitates dispersal between native environments and anthropogenic habitats. Notably, the individual collected from this garden marks the first record from a coastal, unprotected area on Terceira Island, showing the capacity of certain human-modified habitats to support endemic taxa in the region (Tsafack et al. 2021, Boieiro et al. 2025).

Other interesting sampled endemic species were the spider *Emblynaacoreensis* Wunderlich, 1992 (Fig. 3b) and the beetle *Heteroderesazoricus* (Tarnier, 1860) (Fig. 3c), usually common at low elevations in Azores and associated with both native vegetation and exotic trees.

**Figure 4.**
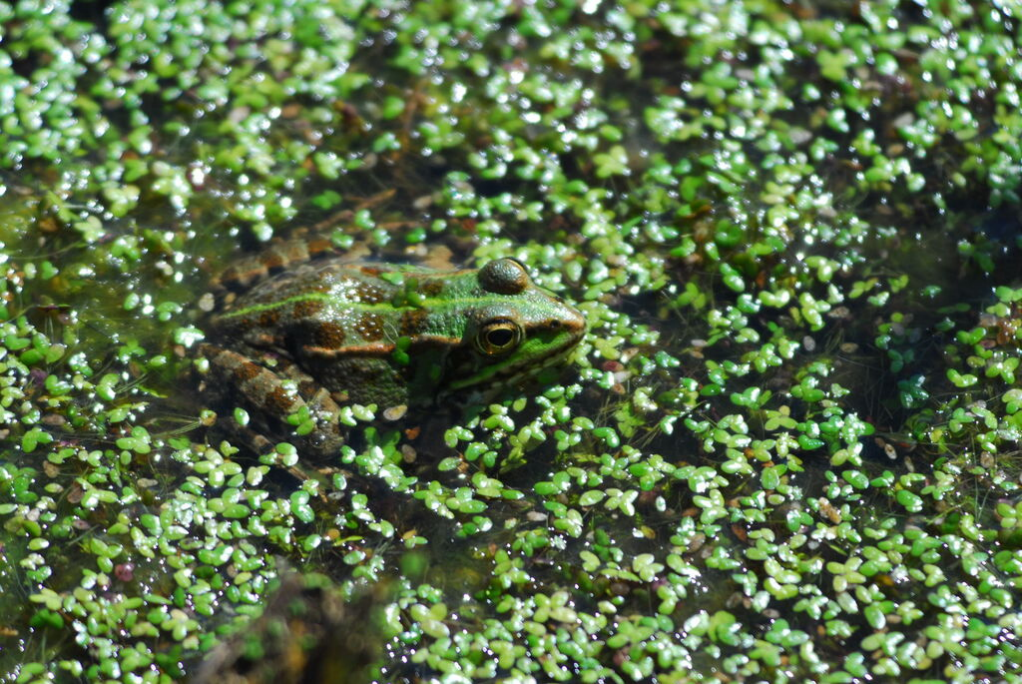
The Iberian water frog *Pelophylaxperezi* (Credit: Pedro Cardoso).

The BioBlitz event on Terceira Island revealed a limited assemblage of introduced freshwater species (Table 4). The survey recorded *Carassiusauratus* (goldfish) from the family Cyprinidae, *Gambusiaholbrooki* (eastern mosquitofish) from the family Poeciliidae and *Pelophylaxperezi* (Iberian water frog) from the family Ranidae (Fig. 4). These non-native species are clear indicators of human-mediated introductions that have not only reshaped the local freshwater ecosystems in the garden, but also signalled broader alterations to Azorean freshwater habitats. Their presence suggests that ongoing anthropogenic activities are influencing habitat composition and dynamics, potentially leading to alterations in native biodiversity and ecosystem processes. This finding emphasises the importance of monitoring and managing invasive species to safeguard the ecological integrity of freshwater systems in the region, a challenge that is increasingly critical in light of rapid environmental changes and urban expansion.

Regarding birds, this event documented 14 taxa (Table 4) (Fig. 5), including seven Azorean endemic subspecies, two native species and five introduced taxa, reflecting a significant endemic presence at lower elevation.

Amongst the larger birds, observed flying over the garden, were two endemic subspecies: *Buteobuteorothschildi* Swann, 1919 (Azores buzzard), a key avian predator in the Archipelago and *Larusmichahellisatlantis* Dwight, 1922 (Atlantic yellow-legged gull), commonly seen patrolling coastal and inland areas. Recorded also were *Columbapalumbusazorica* Hartert, E, 1905 (Azores wood pigeon) (Fig. 5a), an endemic subspecies favouring wooded environments and *Columbalivia* Gmelin, JF, 1789 (rock pigeon), an introduced species often associated with human settlements. The native *Streptopeliadecaocto* (Frivaldszky, 1838) (Eurasian collared dove) was also present. In the garden itself, a variety of passerines were actively feeding and singing. Endemic species included *Motacillacinereapatriciae* Vaurie, 1957 (Azores grey wagtail), *Sturnusvulgarisgranti* Hartert, E, 1903 (Azores common starling), *Sylviaatricapillagularis* Alexander, 1898 (Azores blackcap) (Fig. 5c) and *Turdusmerulaazorensis* Hartert, E, 1905 (Azores blackbird) (Fig. 5d), all of which play vital ecological roles in seed dispersal and insect control. The native *Serinuscanaria* (Linnaeus, 1758) (wild canary) (Fig. 5b), was also present. Introduced passeriforms included *Estrildaastrild* (Linnaeus, 1758) (common waxbill), *Cardueliscarduelisparva* Tschusi, 1901 (European goldfinch), *Chlorischlorisaurantiiventris* (Cabanis, 1851) (European greenfinch) and *Passerdomesticus* (Linnaeus, 1758) (house sparrow), species that have established themselves in the Island’s urban and rural landscapes. Their presence underscores the influence of human-mediated introductions on local avian biodiversity.

### Strengthening the Scientific Contribution

The BioBlitz surveys on Terceira Island provide a valuable opportunity to address critical knowledge gaps in Azorean biodiversity research (Malumbres-Olarte et al. 2019, Amorim et al. 2023, Borges et al. 2023). While the biodiversity of the Azores is well-documented, particularly in natural forested habitats (Borges et al. 2006, Borges et al. 2020, Borges et al. 2022), urban green spaces remain understudied in terms of their potential role in harbouring both native and exotic species. This study helps to bridge that gap by systematically documenting species occurrences in a public garden, an often overlooked habitat in regional biodiversity assessments.

Comparisons with previous biodiversity studies in the Azores suggest that urban gardens, such as "Jardim Duque da Terceira", act as both a source-sink dynamic between habitats, refuges for native biodiversity, while simultaneously serving as entry points for exotic species (Arteaga et al. 2020, Lamelas-López et al. 2023). The findings from our bioblitzes provide additional records that enhance our understanding of species distributions, particularly for lichens, arthropods and vascular plants. Notably, our study confirms the presence of species previously unrecorded in this urban setting, including both recently introduced non-native species and locally rare endemic species.

Preliminary analyses suggest the detection of new or rare species, reinforcing the value of citizen-science initiatives in biodiversity discovery and monitoring. For example, the identification of a rare lichen species in the 2023 BioBlitz suggests that microhabitats within urban gardens may support cryptic biodiversity that has not been well-documented. Additionally, the presence of Azorean endemic arthropods at low elevations (Tsafack et al. 2021) aligns with recent findings that small patches of urban green spaces can provide microclimatic-suitable conditions for native species facing habitat loss.

Importantly, this dataset holds strong potential for long-term biodiversity monitoring. By providing baseline data from 2019 and 2023, this study establishes a foundation for tracking species turnover, population dynamics and invasion processes in future BioBlitz Azores events. Continued monitoring using standardised survey methods could provide information for conservation management strategies, particularly in urban settings where biodiversity is under pressure from habitat fragmentation/destruction and climate change (Ferreira et al. 2016). Moreover, repetitive sampling can provide information on the population dynamics of the area, which is crucial to assess its adequacy as refuge for indigenous species. Future studies should integrate molecular approaches (genetics/genomics) to enhance taxonomic resolution and track genetic shifts in populations over time.

By incorporating this dataset into global biodiversity platforms (GBIF), our findings contribute to broader efforts in data compilation, mobilisation and open-access biodiversity research. The combination of community engagement and rigorous scientific methodology ensures that BioBlitz events remain a valuable tool for both public education and biodiversity conservation in the Azores and beyond.

### Concluding Remarks

The BioBlitz Azores events at "Jardim Duque da Terceira" have provided critical insights into the biodiversity of urban green spaces in the Azores, reinforcing their ecological and conservation value. These surveys highlight the scientific impact of combining citizen science with rigorous taxonomic assessments, demonstrating that even small, anthropogenic habitats can support native, endemic and newly-introduced species. By systematically documenting species richness across multiple taxa — including lichens, vascular plants, arthropods, birds and freshwater vertebrates — this initiative has established a baseline dataset that can be used for future biodiversity monitoring and comparative studies. Importantly, the conservation implications of these findings extend beyond scientific discovery. Urban gardens, such as "Jardim Duque da Terceira", may function as microhabitat refuges for native and endemic species, contributing to the resilience of island biodiversity in the face of habitat loss and climate change. In fact, being an historic garden funded in 1822, "Jardim Duque da Terceira" may be providing suitable habitat for many species for the past two centuries.

Future research will build on this dataset by implementing long-term biodiversity monitoring programmes to track species turnover, population trends and the effects of environmental change in urban and semi-natural habitats. Additional efforts will focus on seasonal and day-time and night-time surveys to capture temporal and daily variation in species assemblages, as well as the application of DNA-based identification techniques to improve taxonomic resolution for cryptic or morphologically challenging taxa (e.g. the case of arthropod morphospecies not yet identified). Expanding BioBlitz Azores to other locations within Terceira Island and, more importantly, to other Azorean Islands will further enhance our understanding of island biogeography, species distributions and conservation needs in human-modified landscapes.

By fostering continued public engagement and integrating citizen science with professional biodiversity assessments, BioBlitz Azores serves as a model for participatory biodiversity conservation, strengthening connections between people and nature, while generating high-quality biodiversity data for research and policy development.

## Figures and Tables

**Figure 2a.**
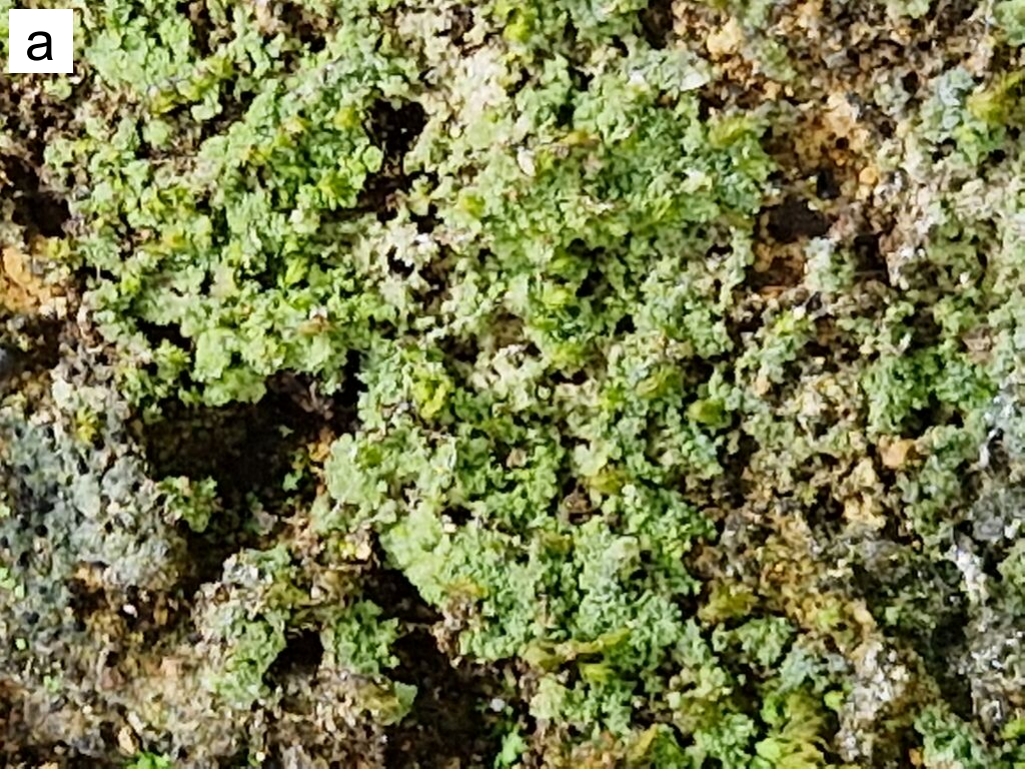
*Botryoleprarialesdainii* (Credit: A.F. Rodrigues);

**Figure 2b.**
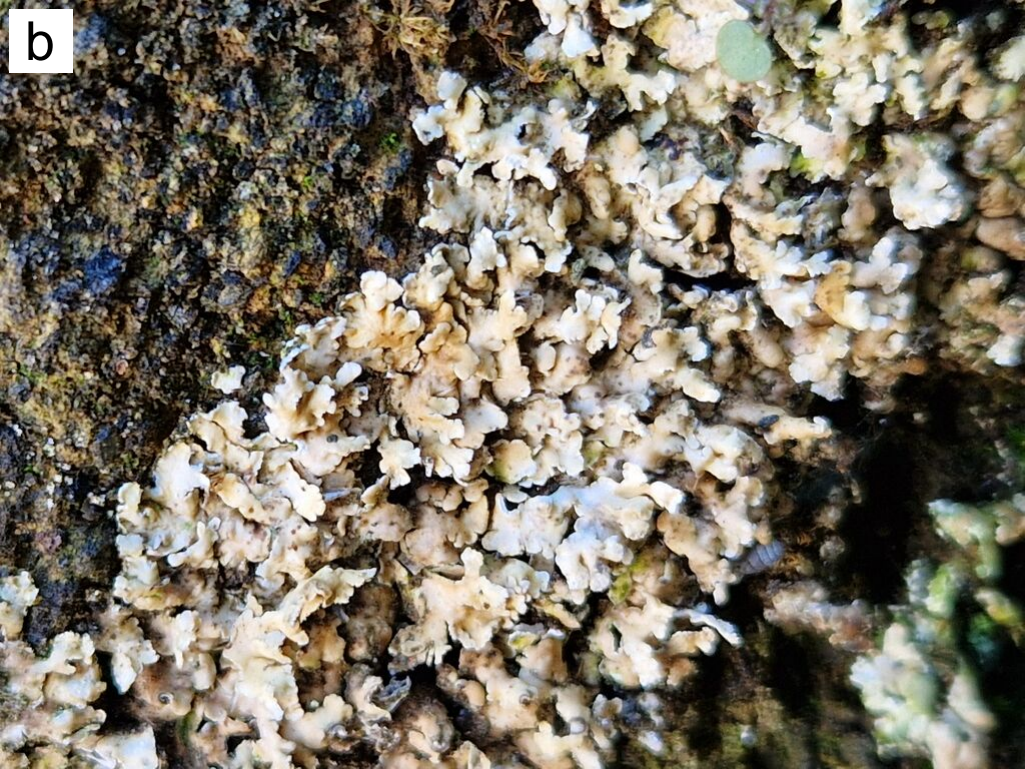
*Hypocenomycescalaris* (Credit: A.F. Rodrigues);

**Figure 2c.**
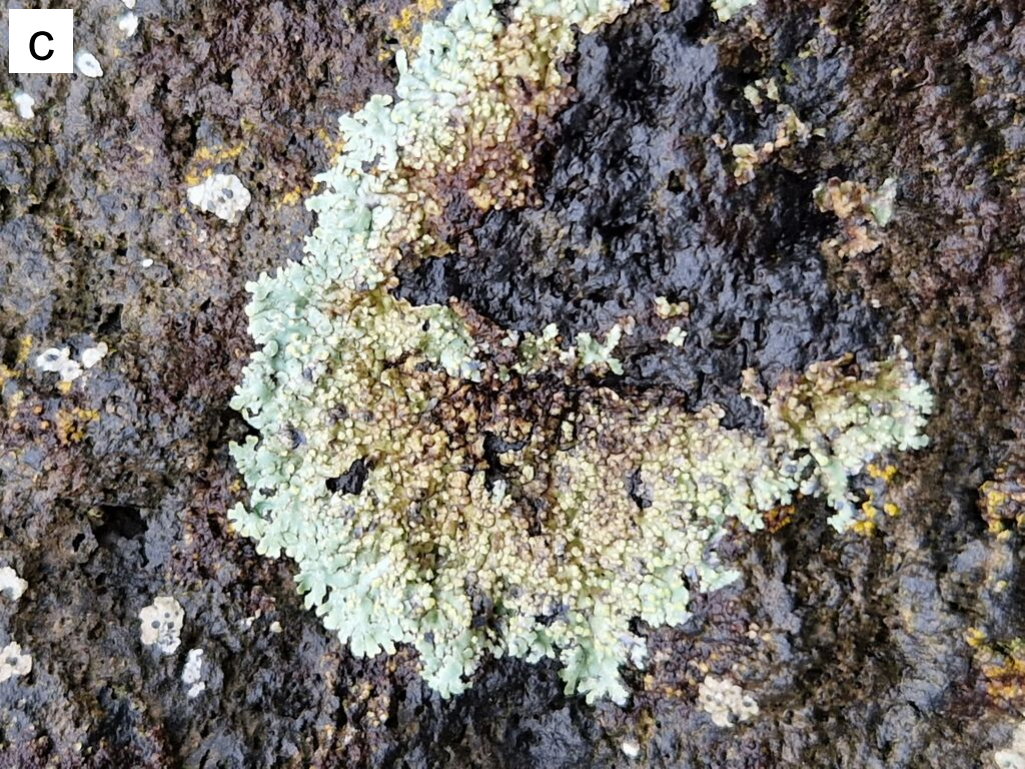
*Hypotrachynarevoluta* (Credit: A.F. Rodrigues);

**Figure 2d.**
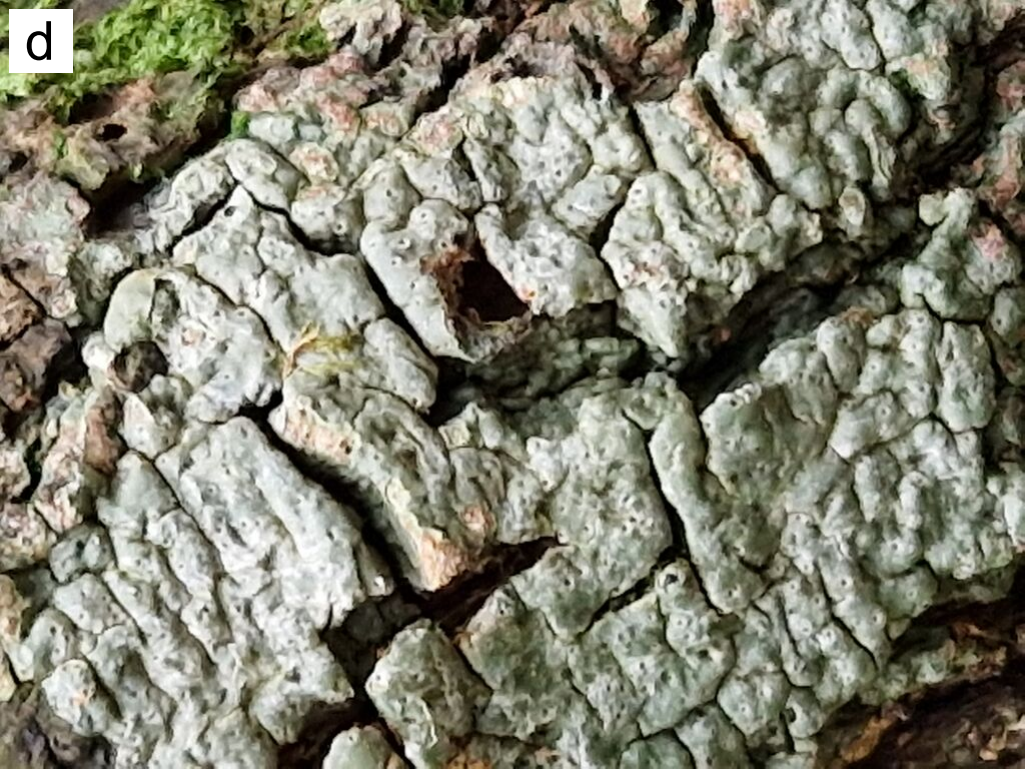
*Pertusariapertusa* (Credit: A.F. Rodrigues);

**Figure 2e.**
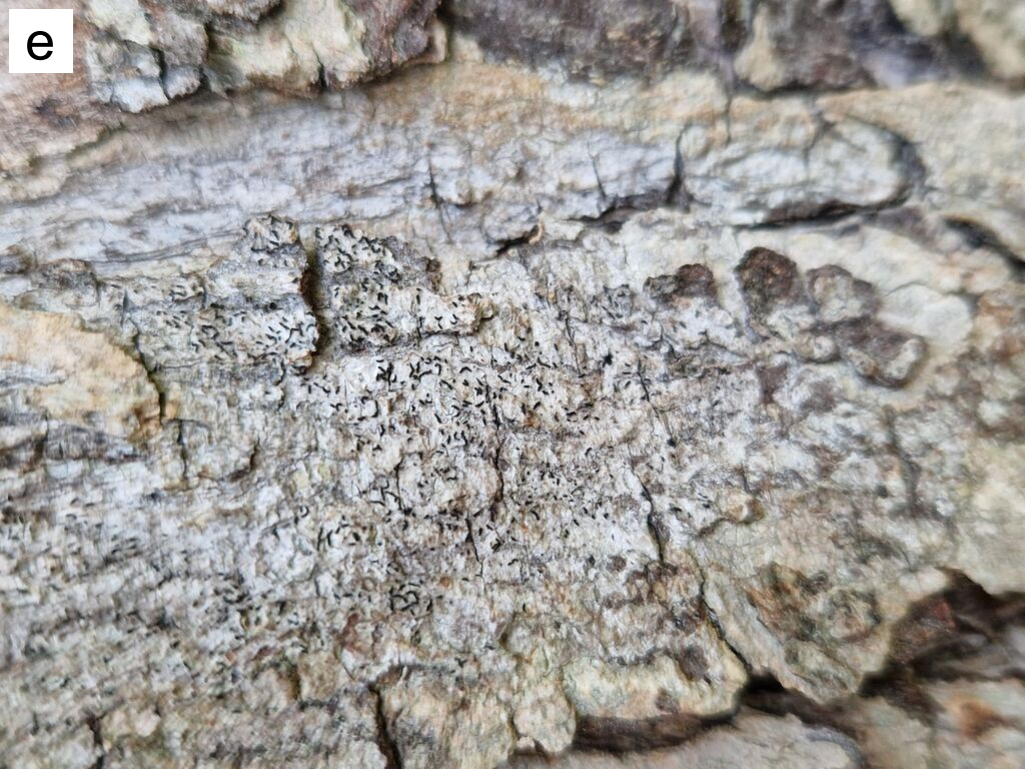
*Pseudoschismatommarufescens* (Credit: A.F. Rodrigues);

**Figure 2f.**
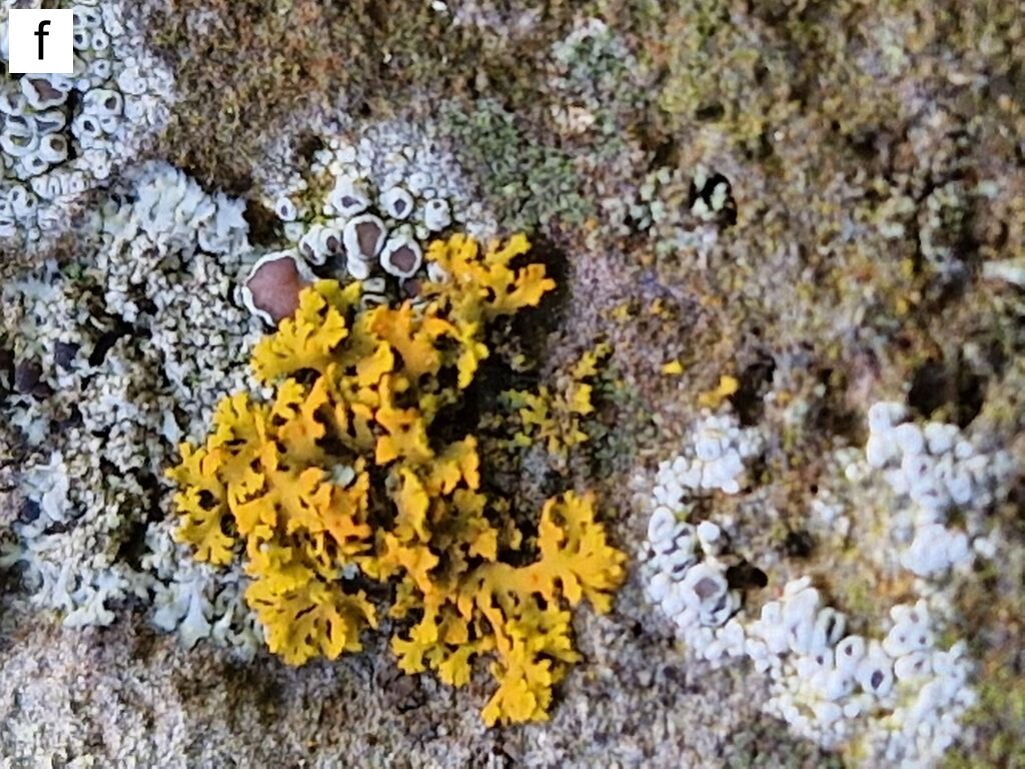
*Polycaulionacandelaria* (Credit: A.F. Rodrigues).

**Figure 3a.**
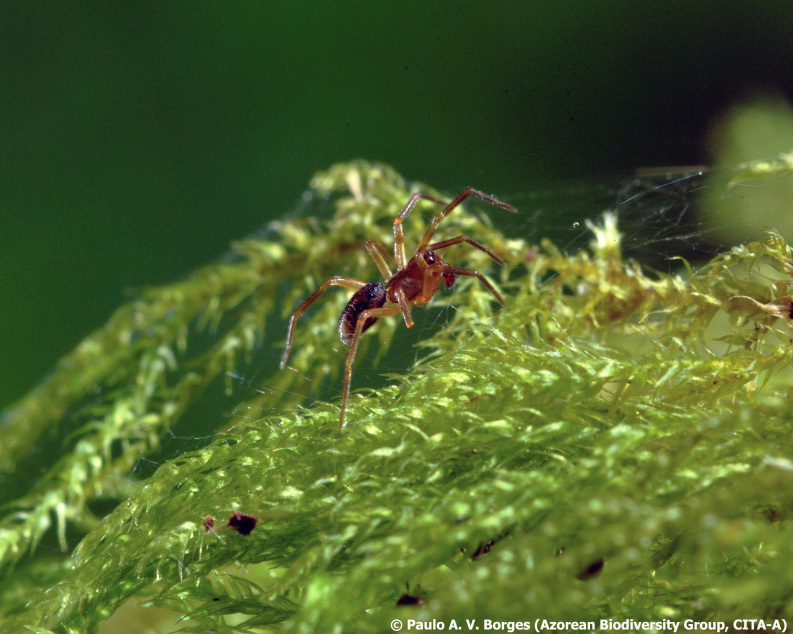
*Savigniorrhipisacoreensis* (Credit: Paulo A. V. Borges);

**Figure 3b.**
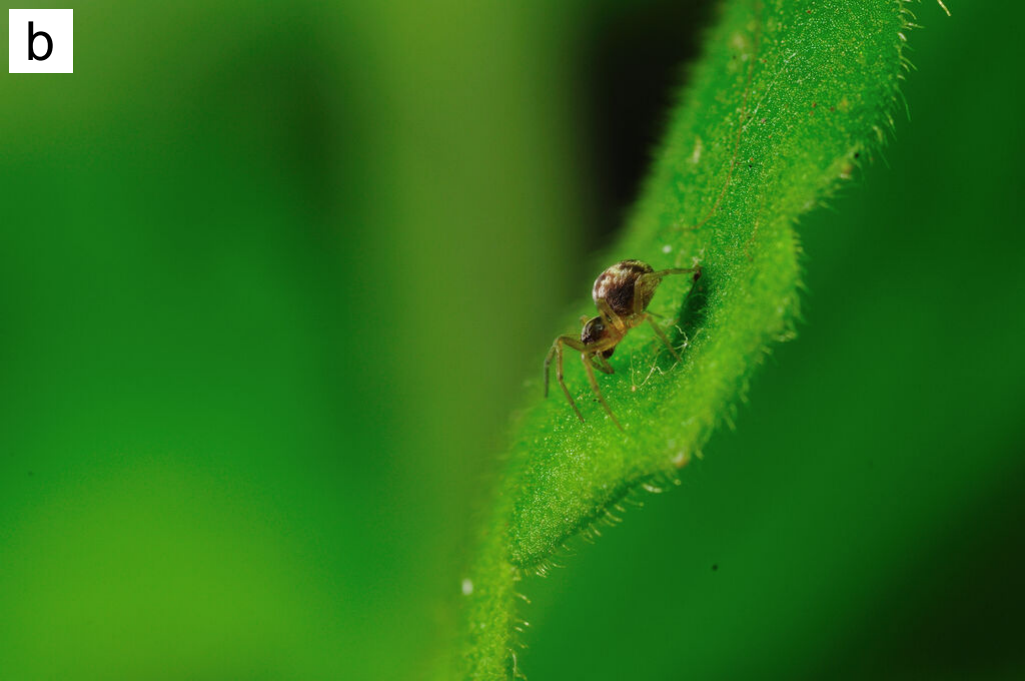
The endemic spider *Emblynaacoreensis* (Credit: Paulo A.V. Borges);

**Figure 3c.**
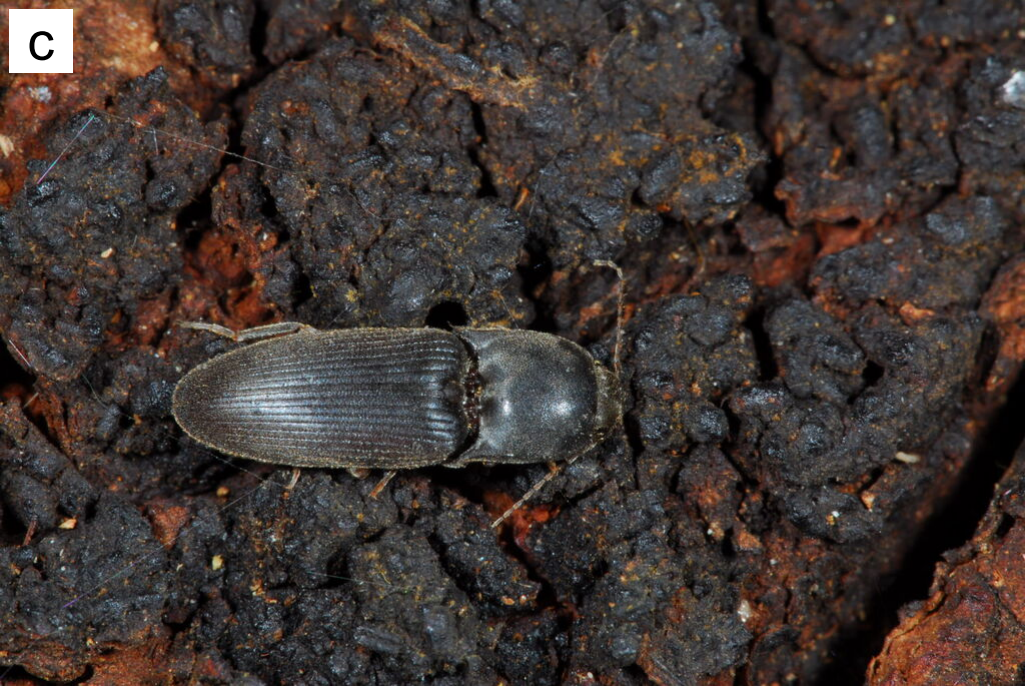
The elaterid beetle *Heteroderesazoricus* (Credit: Pedro Cardoso).

**Figure 5a.**
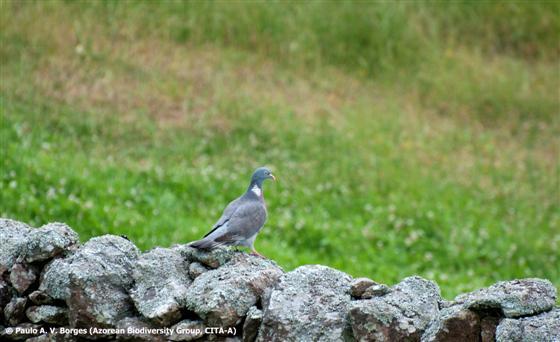
*Columbapalumbisazorenesis* (Credit: Paulo A. V. Borges);

**Figure 5b.**
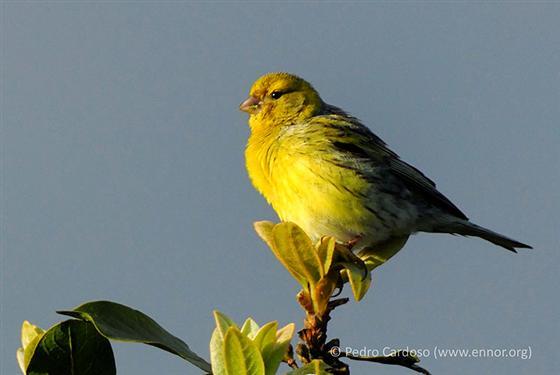
*Serinuscanaria* (Credit: Pedro Cardoso);

**Figure 5c.**
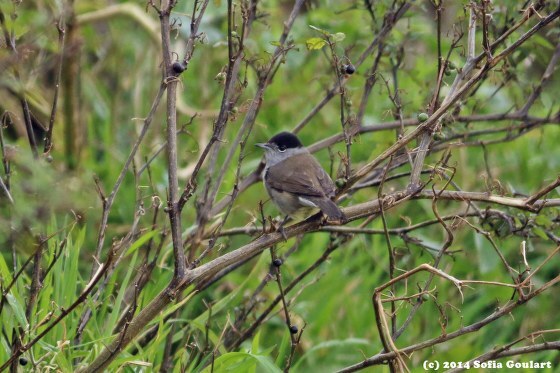
*Sylviaatricapillagularis* (Credit: Sofia Goulart);

**Figure 5d.**
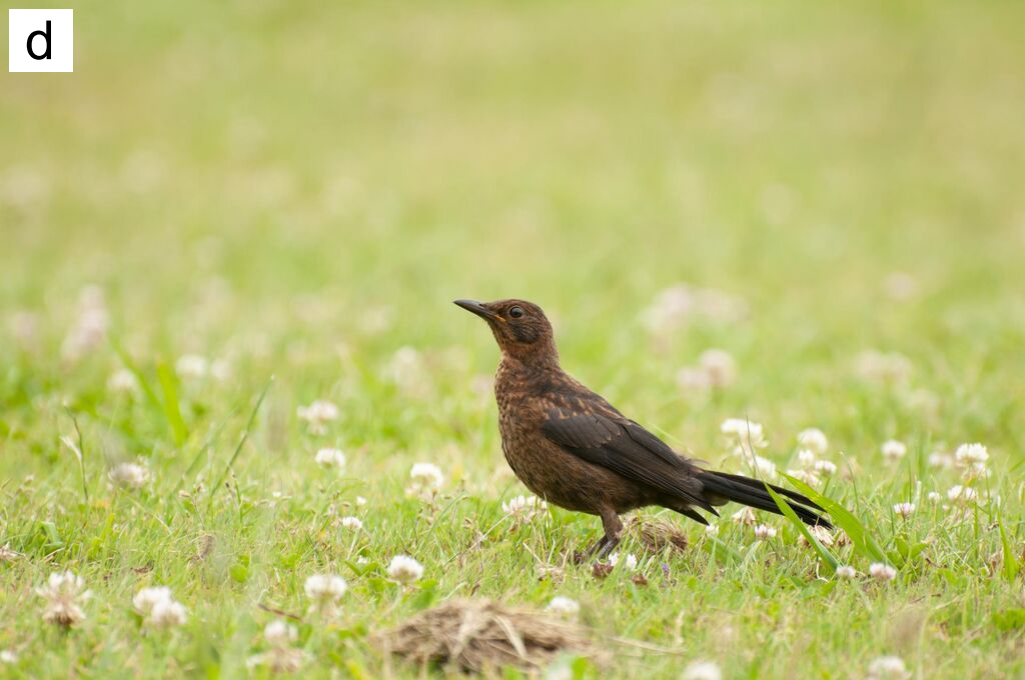
*Turdusmerulaazorensis* (Credit: Paulo A. V. Borges).

**Table 1.** List of lichens found in Public Garden "Jardim Duque da Terceira" (Angra do Heroísmo, Terceira Island).

**Class**	**Order**	**Family**	**Scientific Name**
Arthoniomycetes	Arthoniales	Arthoniaceae	*Arthoniaatra* (Pers.) A.Schneid.
		Chrysotrichaceae	*Chrysothrixcandelaris* (L.) J.R.Laundon
		Lecanographaceae	*Alyxoriavaria* (Pers.) Ertz & Tehler
		Opegraphaceae	*Opegraphavermicellifera* (J.Kunze) J.R.Laundon
			*Opegraphavulgata* (Ach.) Ach.
		Roccellaceae	*Dirinamassiliensis* Durieu & Mont.
			*Enterographacrassa* (DC.) Fée
			*Enterographahutchinsiae* (Leight.) A.Massal.
			*Pseudoschismatommarufescens* (Pers.) Ertz & Tehler
			*Roccellafuciformis* (L.) DC.
			*Roccellatinctoria* DC.
		Roccellographaceae	*Roccellographacircumscripta* (Leight.) Ertz & Tehler
Candelariomycetes	Candelariales	Candelariaceae	*Candelariellavitellina* (Ehrh.) Müll.Arg.
Eurotiomycetes	Verrucariales	incertae sedis	*Botryoleprarialesdainii* (Hue) Canals, Hern.-Mariné, Gómez-Bolea & Llimona
Lecanoromycetes	Caliciales	Caliciaceae	*Amandineapunctata* (Hoffm.) Coppins & Scheid.
			*Buelliadisciformis* (Fr.) Mudd
			*Buelliagriseovirens* (Turner & Borrer ex Sm.) Almb.
			*Buelliasubdisciformis* (Leight.) Vain.
			*Diploiciacanescens* (Dicks.) A.Massal.
			*Diplotommaalboatrum* (Hoffm.) Flot.
			*Diplotommaambiguum* (Ach.) Flagey
			*Dirinariaapplanata* (Fée) D.D.Awasthi
			*Pyxinesorediata* (Ach.) Mont.
			*Pyxinesubcinerea* Stirt.
		Physciaceae	*Hyperphysciaadglutinata* (Flörke) H.Mayrhofer & Poelt
			*Physciacaesia* (Hoffm.) Fürnr.
			*Physciadimidiata* (Arnold) Nyl.
			*Polyblastidiumalbicans* (Pers.) S.Y. Kondr., Lőkös & Hur
	Graphidales	Graphidaceae	*Graphisscripta* (L.) Ach.
	Lecanorales	Cladoniaceae	*Cladoniachlorophaea* (Flörke ex Sommerf.) Spreng.
			*Cladoniaochrochlora* Flörke
			*Hertelianagagei* (Sm.) J.R.Laundon
			*Leprariaincana* (L.) Ach.
			*Leprarialobificans* Nyl.
			*Leprariamembranacea* (Dicks.) Vain.
		Lecanoraceae	*Carbonicolaanthracophila* (Nyl.) Bendiksby & Timdal
			*Lecanoracampestris* (Schaer.) Hue
			*Lecanoracenisia* Ach.
			*Lecanorachlarotera* Nyl.
			*Myriolecisdispersa* (Pers.) Śliwa, Zhao Xin & Lumbsch
			*Protoparmeliopsismuralis* (Schreb.) M.Choisy
			*Pyrrhosporaquernea* (Dicks.) Körb.
		Parmeliaceae	*Hypotrachynarevoluta* (Flörke) Hale
			*Parmotremareticulatum* (Taylor) M.Choisy
			*Parmotremarobustum* (Degel.) Hale
			*Parmotrematinctorum* (Despr. ex Nyl.) Hale
		Ramalinaceae	*Bacidiaarceutina* (Ach.) Arnold
			*Bacidialaurocerasi* (Delise ex Duby) Zahlbr.
			*Biatoraefflorescens* (Hedl.) Räsänan
			*Ramalinabourgaeana* Mont. ex Nyl.
			*Ramalinafarinacea* (L.) Ach.
			*Ramalinalusitanica* H.Magn.
			*Ramalinarequienii* (De Not.) Jatta
		Tephromelataceae	*Mycoblastusaffinis* (Schaer.) T.Schauer
			*Tephromelaatra* (Huds.) Hafellner
	Lecideales	Lecideaceae	*Clauzadeaimmersa* (Hoffm.) Hafellner & Bellem.
	Peltigerales	Collemataceae	*Blennothalliacrispa* (Hudson) Otálora, P.M.Jørg. & Wedin
			*Collemafurfuraceum* (Arnold) Du Rietz
			*Collemasubflaccidum* Degel.
			*Enchyliumtenax* (Sw.) Gray
		Pannariaceae	*Fuscopannarianebulosa* (Hoffm.) E.Tripp & Lendemer
	Pertusariales	Ochrolechiaceae	*Ochrolechiaandrogyna* (Hoffm.) Arnold
		Pertusariaceae	*Pertusariahymenea* (Ach.) Schaer.
			*Pertusariapertusa* (L.) Tuck.
			*Verseghyathysanophora* (R.C.Harris) S.Y.Kondr.
		Variolariaceae	*Lepraamara* (Ach.) Hafellner
	Teloschistales	Teloschistaceae	*Caloplacadalmatica* (A. Massal.) H.Olivier
			*Gyalolechiaflavorubescens* (Huds.) Søchting, Frödén & Arup
			*Polycaulionacandelaria* (Linnaeus) Frödén, Arup & Søchting
			*Variosporaflavescens* (Huds.) Arup, Frödén & Søchting
			*Xanthoriaparietina* (L.) Th.Fr.
	Umbilicariales	Ophioparmaceae	*Hypocenomycescalaris* (Ach. ex Lilj.) M.Choisy

**Table 2.** List of the identified Vascular Plants. The several Phyla are in bold.

**Phylum/Class**	**Order**	**Family**	**Scientific Name**	**Colonisation** **Status**
**Ginkgophyta**				
Ginkgoopsida	Ginkgoales	Ginkgoaceae	*Ginkgobiloba* L.	introduced
**Magnoliophyta**				
Liliopsida	Alismatales	Araceae	*Monsteradeliciosa* Liebm.	introduced
	Arecales	Arecaceae	*Phoenixcanariensis* H.Wildpret	introduced
	Asparagales	Amaryllidaceae	*Agapanthusafricanus* Hoffmanns.	introduced
		Asparagaceae	*Agaveattenuata* Salm-Dyck	introduced
			*Asparagusdensiflorus* (Kunth) Jessop	introduced
			*Chlorophytumcomosum* (Thunb.) Jacques	introduced
			*Dracaenadraco* (L.) L.	indeterminate
		Asphodelaceae	*Aloearborescens* Mill.	introduced
	Commelinales	Pontederiaceae	*Eichhorniacrassipes* Solms	introduced
	Poales	Cyperaceae	*Cyperuspapyrus* L.	introduced
		Poaceae	*Festucaglauca* Vill.	introduced
			*Festucapetraea* Guthnick ex Seub.	endemic
	Zingiberales	Cannaceae	*Cannaindica* L.	introduced
		Strelitziaceae	*Strelitzianicolai* Regel & K.Koch	introduced
			*Strelitziareginae* Banks	introduced
Magnoliopsida	Apiales	Apiaceae	*Scheffleraarboricola* (Hayata) Merr.	introduced
	Aquifoliales	Aquifoliaceae	*Ilexperado* Soland. ex Aiton	introduced
	Ericales	Ericaceae	*Rhododendronindicum* Sweet	introduced
		Theaceae	*Camelliajaponica* L.	introduced
	Fabales	Fabaceae	*Ceratoniasiliqua* L.	introduced
			*Trifoliumrepens* L.	introduced
			*Wisteriasinensis* Sweet	introduced
	Gentianales	Apocynaceae	*Neriumoleander* L.	introduced
			*Plumeriarubra* L.	introduced
		Rubiaceae	*Coffeaarabica* L.	introduced
			*Coprosmarepens* A.Rich.	introduced
	Lamiales	Lamiaceae	*Lavanduladentata* L.	introduced
	Laurales	Lauraceae	*Cinnamomumcamphora* (L.) J.Presl	introduced
			*Perseaamericana* Mill.	introduced
			*Phoebeindica* Pax	introduced
	Magnoliales	Magnoliaceae	*Liriodendrontulipifera* L.	introduced
			*Magnoliagrandiflora* L.	introduced
	Malpighiales	Euphorbiaceae	*Acalyphawilkesiana* Mull.Arg.	introduced
	Malvales	Malvaceae	*Brachychitonacerifolius* F.Muell.	introduced
			*Ceibaspeciosa* (A.St.-Hil., A.Juss. & Cambess.) Ravenna	introduced
			*Hibiscusrosa-sinensis* L.	introduced
			*Hibiscussyriacus* L.	introduced
			*Tiliacordata* Mill.	introduced
	Myrtales	Lythraceae	*Lagerstroemiaindica* L.	introduced
		Myrtaceae	*Corymbiacitriodora* (Hook.) K.D.Hill & L.A.S.Johnson	introduced
			*Eugeniauniflora* L.	introduced
			*Metrosiderosexcelsa* Gaertn.	introduced
	Nymphaeales	Nymphaeaceae	*Nymphaeaalba* L.	introduced
	Rosales	Moraceae	*Ficusmicrocarpa* L.f.	introduced
			*Ficuspumila* L.	introduced
			*Morusnigra* L.	introduced
	Solanales	Solanaceae	*Brugmansiasuaveolens* Bercht. & J.Presl	introduced
**Pinophyta**				
Pinopsida	Pinales	Araucariaceae	*Araucariaheterophylla* (Salisb.) Franco	introduced
		Podocarpaceae	*Podocarpusmacrophyllus* Sweet	introduced
**Pteridophyta**				
Polypodiopsida	Cyatheales	Cyatheaceae	*Sphaeropteriscooperi* (F. Muell.) R.M.Tryon	introduced
	Polypodiales	Pteridaceae	*Adiantumcapillus-veneris* L.	native

**Table 3.** List of identified arthropods at species or subspecies level.

**Class**	**Order**	**Family**	**Scientific Name**	**Colonisation Status**
Arachnida	Araneae	Araneidae	*Agalenatearedii* (Scopoli, 1763)	introduced
			*Araneusangulatus* Clerck, 1757	introduced
			*Argiopebruennichi* (Scopoli, 1772)	native
			*Mangoraacalypha* (Walckenaer, 1802)	introduced
			*Neosconacrucifera* (Lucas, 1838)	introduced
		Cheiracanthiidae	*Cheiracanthiummildei* L. Koch, 1864	introduced
		Clubionidae	*Porrhoclubionadecora* (Blackwall, 1859)	native
		Dictynidae	*Emblynaacoreensis* Wunderlich, 1992	endemic
			*Nigmapuella* (Simon, 1870)	introduced
		Linyphiidae	*Erigoneautumnalis* Emerton, 1882	introduced
			*Mermessusbryantae* (Ivie & Barrows, 1935)	introduced
			*Mermessusfradeorum* (Berland, 1932)	introduced
			*Savigniorrhipisacoreensis* Wunderlich, 1992	endemic
			*Tenuiphantestenuis* (Blackwall, 1852)	introduced
		Mimetidae	*Eroaphana* (Walckenaer, 1802)	introduced
		Salticidae	*Heliophanuskochii* Simon, 1868	introduced
			*Macaroerisdiligens* (Blackwall, 1867)	native
			*Pseudeuophrysvafra* (Blackwall, 1867)	introduced
			*Salticusmutabilis* Lucas, 1846	introduced
		Tetragnathidae	*Metellinamerianae* (Scopoli, 1763)	introduced
		Theridiidae	*Cryptachaeablattea* (Urquhart, 1886)	introduced
			*Paidiscuraorotavensis* (Schmidt, 1968)	native
			*Steatodanobilis* (Thorell, 1875)	native
Diplopoda	Julida	Julidae	*Ommatoiulusmoreleti* (Lucas, 1860)	introduced
Insecta	Coleoptera	Apionidae	*Aspidapionradiolus* (Marsham, 1802)	introduced
			*Kalcapionsemivittatumsemivittatum* (Gyllenhal, 1833)	indeterminate
		Chrysomelidae	*Longitarsuskutscherai* (Rye, 1872)	introduced
		Coccinellidae	*Clitostethusarcuatus* (Rossi, 1794)	introduced
			*Noviuscardinalis* (Mulsant, 1850)	introduced
			*Rhyzobiuslophanthae* (Blaisdell, 1892)	introduced
			*Scymniscushelgae* (Fürsch, 1965)	introduced
			*Scymnusinterruptus* (Goeze, 1777)	native
			*Stethoruspusillus* (Herbst, 1797)	native
		Corylophidae	*Sericoderuslateralis* (Gyllenhal, 1827)	introduced
		Curculionidae	*Coccotrypescarpophagus* (Hornung, 1842)	introduced
			*Lixuspulverulentus* (Scopoli, 1763)	introduced
			*Naupactuscervinus* (Boheman, 1840)	introduced
			*Sitonadiscoideus* Gyllenhal, 1834	introduced
		Elateridae	*Heteroderesazoricus* (Tarnier, 1860)	endemic
			*Heteroderesvagus* Candèze, 1893	introduced
		Nitidulidae	*Brassicogethesaeneus* (Fabricius, 1775)	introduced
			*Carpophilusfumatus* Boheman, 1851	introduced
		Phalacridae	*Stilbustestaceus* (Panzer, 1797)	native
		Silvanidae	*Cryptamorphadesjardinsii* (Guérin-Méneville, 1844)	introduced
		Staphylinidae	*Carpelimuszealandicus* (Sharp, 1900)	introduced
	Hemiptera	Cicadellidae	*Anoscopusalbifrons* (Linnaeus, 1758)	native
			*Euscelidiusvariegatus* (Kirschbaum, 1858)	native
			*Sophoniaorientalis* (Matsumura, 1912)	introduced
		Delphacidae	*Kelisiaribauti* Wagner, 1938	native
		Flatidae	*Siphantaacuta* (Walker, 1851)	introduced
		Miridae	*Heterotomaplanicornis* (Pallas, 1772)	native
			*Pilophorusconfusus* (Kirschbaum, 1856)	native
			*Pilophorusperplexus* Douglas & Scott, 1875	native
			*Taylorilygusapicalis* (Fieber, 1861)	introduced
			*Trigonotyluscaelestialium* (Kirkaldy, 1902)	native
		Nabidae	*Nabispseudoferusibericus* Remane, 1962	native
		Reduviidae	*Ploiariachilensis* (Philippi, 1862)	introduced
		Rhyparochromidae	*Heterogasterurticae* (Fabricius, 1775)	native
			*Scolopostethusdecoratus* (Hahn, 1833)	native
		Triozidae	*Triozalaurisilvae* Hodkinson, 1990	native
	Hymenoptera	Formicidae	*Hypoponeraeduardi* (Forel, 1894)	native
			*Lasiusgrandis* Forel, 1909	native
			*Linepithemahumile* (Mayr, 1868)	introduced
			*Monomoriumcarbonarium* (Smith, 1858)	native
			*Tetramoriumcaespitum* (Linnaeus, 1758)	native
			*Tetramoriumcaldarium* (Roger, 1857)	introduced
	Lepidoptera	Noctuidae	*Autographagamma* (Linnaeus, 1758)	native
		Tineidae	*Oinophilav-flava* (Haworth, 1828)	introduced
	Neuroptera	Chrysopidae	*Chrysoperlalucasina* (Lacroix, 1912)	introduced
	Odonata	Aeshnidae	*Anaximperator* Leach, 1815	native
	Orthoptera	Tettigoniidae	*Phaneropteranana* Fieber, 1853	native
	Psocodea	Caeciliusidae	*Valenzuelaburmeisteri* (Brauer, 1876)	native
			*Valenzuelaflavidus* (Stephens, 1836)	native
		Ectopsocidae	*Ectopsocusbriggsi* McLachlan, 1899	introduced
			*Ectopsocusstrauchi* Enderlein, 1906	native
		Epipsocidae	*Bertkauialucifuga* (Rambur, 1842)	native
		Trichopsocidae	*Trichopsocusclarus* (Banks, 1908)	native
	Thysanoptera	Phlaeothripidae	*Hoplothripscorticis* (De Geer, 1773)	native

**Table 4.** The list of identified Chordata.

**Class**	**Order**	**Family**	**Scientific Name**	**Colonisation status**
Actinopterygii	Cypriniformes	Cyprinidae	*Carassiusauratus* (Linnaeus, 1758)	introduced
		Poecilidae	*Gambusiaholbrooki* Girard, 1859	introduced
Amphibia	Anura	Ranidae	*Pelophylaxperezi* (López-Seoane, 1885)	introduced
Aves	Accipitriformes	Accipitridae	*Buteobuteorothschildi* Swann, 1919	endemic
	Charadriiformes	Laridae	*Larusmichahellisatlantis* Dwight, 1922	endemic
	Columbiformes	Columbidae	*Columbalivia* Gmelin, JF, 1789	introduced
			*Columbapalumbusazorica* Hartert, E, 1905	endemic
			*Streptopeliadecaocto* (Frivaldszky, 1838)	native
	Passeriformes	Estrildidae	*Estrildaastrild* (Linnaeus, 1758)	introduced
		Fringillidae	*Cardueliscarduelisparva* Tschusi, 1901	introduced
			*Chlorischlorisaurantiiventris* (Cabanis, 1851)	introduced
			*Serinuscanaria* (Linnaeus, 1758)	native
		Motacillidae	*Motacillacinereapatriciae* Vaurie, 1957	endemic
		Passeridae	*Passerdomesticus* (Linnaeus, 1758)	introduced
		Sturnidae	*Sturnusvulgarisgranti* Hartert, E, 1903	endemic
		Sylviidae	*Sylviaatricapillagularis* Alexander, 1898	endemic
		Turdidae	*Turdusmerulaazorensis* Hartert, E, 1905	endemic
